# Experimental and Numerical Investigation on the Shear Behavior of Engineered Cementitious Composite Beams with Hybrid Fibers

**DOI:** 10.3390/ma15145059

**Published:** 2022-07-20

**Authors:** Jeyaprakash Maheswaran, Maheswaran Chellapandian, Madappa V. R. Sivasubramanian, Gunasekaran Murali, Nikolai Ivanovich Vatin

**Affiliations:** 1 Department of Civil Engineering, St. Xavier’s Catholic College of Engineering, Nagercoil 629003, India; drjmaheswaran@gmail.com; 2 Department of Civil Engineering, Mepco Schlenk Engineering College, Sivakasi 626005, India; 3 Department of Civil Engineering, National Institute of Technology, Puducherry 609609, India; madappa@nitpy.ac.in; 4 Peter the Great St. Petersburg Polytechnic University, 195251 Saint Petersburg, Russia; vatin@mail.ru; 5 Division of Research & Innovation, Uttaranchal University, Dehradun 248007, India

**Keywords:** shear behavior, engineered cementitious composites (ECC), hybrid fibers, finite element analysis, PVA fibers, polypropylene fibers

## Abstract

The shear behavior of innovative engineered cementitious composites (ECC) members with a hybrid mix of polyvinyl alcohol (PVA) and polypropylene (PP) fibers is examined. The overall objective of the investigation is to understand the shear behavior of ECC beams with different mono and hybrid fiber combinations without compromising the strength and ductility. Four different configurations of beams were prepared and tested, including 2.0% of PP fibers, 2.0% of PVA fibers, 2.0% of steel fibers and hybrid PVA and PP fibers (i.e., 1% PP and 1% PVA). In addition to the tests, a detailed nonlinear finite element (FE) analysis was accomplished using the commercial ABAQUS software. The validated FE model was used to perform an extensive parametric investigation to optimize the design parameters for the hybrid-fiber-reinforced ECC beams under shear. The results revealed that the use of hybrid PVA and PP fibers improved the performance by enhancing the overall strength and ductility compared to the steel and PP-fiber-based ECC beams. Incorporating hybrid fibers into ECC beams increased the critical shear crack angle, indicating the transition of a failure from a brittle diagonal tension to a ductile bending.

## 1. Introduction

Conventional cement-based concrete is often limited in its applications due to its fragile failure nature and low tensile strength. For several applications in the construction industry, high-strength concrete is mainly preferred [1]. However, this further increases the possibility of catastrophic failures due to its highly brittle nature. Hence, developing an advanced cementitious system with high ductility to the structures is essential [2,3,4,5,6,7,8]. Specifically, the ductile nature is paramount for designing reinforced concrete (RC) structures in seismic-prone areas. In order to achieve the above-specified criteria, an advanced high-performance ductile composite system called engineered cementitious composites (ECC) was used. This composite was developed in the early 1990s and is also recognized as bendable concrete or strain-hardening cementitious composites (SHCC) and can be easily manufactured for practical applications [9,10]. ECC has gained popularity because of its ability to undergo large strain levels before failure [11]. The ultimate strain level of ECC is 3–7%, which is significantly larger than the normal cement concrete [12,13]. Different types of discrete fibers, namely polyethylene (PE), polypropylene, polyvinyl alcohol (PVA) and steel, are randomly dispersed in the ECC matrix to enhance the tensile strength and achieve a ductile failure mechanism [14,15,16].

The shear behavior of ECC elements is influenced by parameters such as (a) the diameter and spacing of transverse reinforcements, (b) shear span (a_v_) to depth (h) ratio, (c) mode of failure and (d) size effect [17,18]. Several preceding works have investigated the ECC-based structural elements’ performance under shear loads [19,20,21,22,23,24,25,26]. Meng et al. [19] examined the shear resistance of polyvinyl alcohol fiber-based ECC members with and without transverse reinforcements. The findings indicated that the development of PVA-based fibers in the ECC matrix helped in enhancing the ductility of ECC beams without stirrups. Moreover, the PVA fiber addition aided in changing the mode of failure from diagonal shear–tension mode to ductile flexure type, even in the absence of stirrups. Alyousif et al. [20] investigated the shear behavior of ECC beams with varying a_v_/h ratios. Results revealed that the inclusion of discrete PVA fibers (*V_f_* = 2.0%) helped in eliminating the necessity of transverse stirrups at the location of critical shear span. Moreover, the strength and ductility of ECC beams increased significantly compared to the regular RC beam. The above observations were not witnessed for ECC members with a higher a_v_/h ratio. When the a_v_/h ratio is increased, the overall load-deflection capacity of ECC beams shows a considerable reduction. The reduction in stiffness and ductility can be ascribed to the shift in the failure type of PVA-based ECC beams from flexure to brittle shear–tension mode. Paegle and Fischer [21] compared the ECC and RC beams’ performance with the transverse reinforcements. The overall performance of ECC beams was superior due to the stirrups and the presence of discrete fibers. The use of discrete fibers in ECC beams helped to activate the stirrups at smaller crack levels through the unique crack bridging mechanism, thereby ensuring stabilized failure progression at a gradual phase.

Ismail and Hassan [22] explored the feasibility of different discrete fiber additions on the shear performance of ECC elements. The findings revealed that using steel and PVA-based fibers in ECC elements helped to compensate for the absence of conventional transverse reinforcements. The previous studies show that the ECC members prepared using different fibers experienced failure due to the ductile flexure mode when loaded in shear [23,24]. Reviewing the existing literature studies, it is clear that only a handful of studies are available for understanding the shear behavior of ECC-based structural elements [25,26,27]. It is worth noting that the use of combined synthetic PVA and PP fibers on the shear behavior of ECC beams is not available. Moreover, the research on the shear performance of ECC members with PP and steel fibers is limited. Hence, the present research work can help to fill the existing knowledge gap by experimentally studying the shear behavior of ECC members with different fiber combinations. In addition to the experimental studies, a detailed finite element analysis was performed for optimizing different design parameters of ECC beams with mono and hybrid fibers under shear.

## 2. Research Significance

The addition of synthetic fibers (i.e., PVA, PP, etc.) could help to improve the serviceability and deformability characteristics of engineered cementitious composite beams. Under shear dominating forces, using discrete fibers can help to eliminate the requirement for transverse reinforcement. It is worth mentioning that no studies in the past have investigated the effectiveness of hybrid fiber addition on the overall behavior of ECC beams under shear. Hence, the main objective of the proposed work is to explore the shear performance of mono and hybrid-fiber-reinforced engineered cementitious composite members. The following are the distinct contributions made through this work.

(a) Quantifying the effect of different types of fiber addition (i.e., mono and hybrid combinations) for improving the overall performance of ECC beams under shear.
(b) Developing a valid nonlinear finite element modeling approach for the structural evaluation of mono and hybrid-fiber-reinforced ECC beams under shear.
(c) Optimizing the different design parameters, namely (a) compressive strength of ECC, (b) ratio of longitudinal reinforcement and (c) transverse reinforcement spacing for hybrid-fiber-based ECC members.

## 3. Experimental Program

### 3.1. Composition of Mix and Materials

All of the ECC members designed as a part of this work were cast at the structural element testing center, Mepco Schlenk Engineering College Sivakasi. The beams were prepared using the ECC design mix procedure specified in the existing literature works to achieve a mean strength of 30 MPa in compression. The mix quantities used for preparing ECC beams are (in kg/m^3^): fly ash = 798; cement = 638; fine aggregate = 539; water = 431; and admixture = 14. Three different fibers—steel, polyvinyl alcohol (PVA) and polypropylene (PP)—were used in this work. The characteristics of the fibers used in this work were obtained from the manufacturer specifications and shown in Table 1. In general, the addition of discrete fibers could retard the workability of concrete mix to a significant extent [22]. Hence, a high-range water-reducing admixture (i.e., Poly-carboxylate-based Conplast SP430) was used to improve the workability of the prepared ECC mix. The fresh property of ECC mix was determined using the slump cone test. Adding steel and PP fibers to ECC mix significantly reduced the workability. However, the ECC mix’s slump values were improved by combining PP and PVA fibers.

### 3.2. Details of Specimens

Eight reinforced ECC members were tested under a three-point bending configuration to evaluate the shear resistance of different hybrid and mono fiber combinations. The parameters assessed in this work include mono and hybrid fiber combinations. The test program includes four different specimen series: (a) ECC beam with 2.0% steel fibers, (b) ECC beam with 2.0% PVA fibers, (c) ECC beam with 2.0% PP fibers and (d) ECC beam with hybrid PP and PVA fibers (Table 2). The fiber volume fraction (*V_f_*) value was kept constant at 2.0% for all of the test specimens to adhere to the minimum dosage amount for standard ECC. Keeping the fiber volume fraction constant, four different combinations of mono and hybrid-fiber-based ECC were investigated. Investigation of ECC with high fiber dosage of more than 2% will be interesting and is the scope for further work. The other important parameters, such as compressive strength, a_v_/d ratio and longitudinal ratio, were kept unchanged to apprehend the effectiveness of hybrid fiber addition on the shear load capacity of ECC.

**Figure 1:**
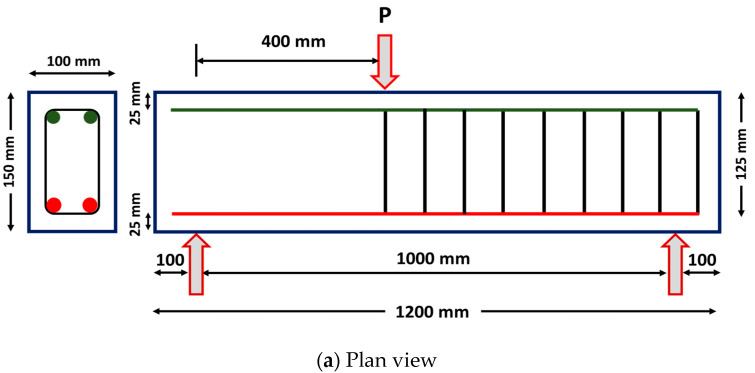

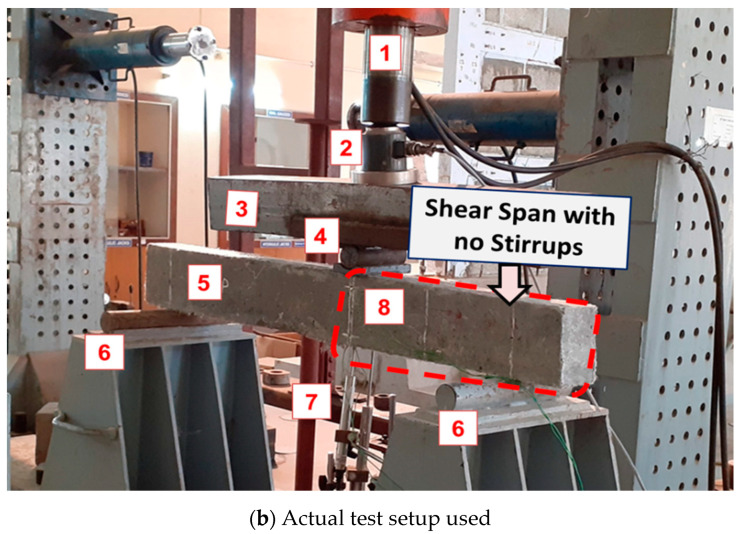
(**a**) Sectional details of the test specimen, (**b**) experimental test setup. Components: 1. A 500 kN Hydraulic Jack, 2. Load Cell, 3. Spreader Beam, 4. Load Point, 5. Test Specimen, 6. Supports, 7. LVDTs for Displacement Measurement and 8. Strain Gauges.

The reinforcement details of the test section are depicted in Figure 1. All of the members had a rectangular cross-section of dimensions 100 mm (b) × 150 mm (d) × 1200 mm (L). The beams were designed to have a shear span (a_v_) value of 400 mm. A low a_v_/d ratio value of 3.2 was designated to ensure dominant shear failure. All of the beams were reinforced with two 10 mm diameter longitudinal reinforcements on the tension side (*A_st_* = 157 mm^2^ and *ρ_s_* = 1.04%). Two steel bars with a 10 mm diameter were provided for holding the stirrups in the compression face of the test specimen. The shear span of the test specimen did not have any transverse reinforcements (stirrups) to localize the dominant shear failure in this test zone. On the other hand, the steel stirrups of 8 mm diameter were provided in the non-test zone (i.e., outside the shear span region) at a spacing of 75 mm c/c to localize the failure within the shear span. A clear cover of 25 mm was provided on all of the faces of the test specimens. The mechanical properties of cast ECC specimens with different fiber dosages were assessed by testing standard cubes of size 150 mm and cylinders of dimensions 150 mm × 300 mm. In order to ensure consistency in test results, a minimum of five samples were tested under compression and the average strength was used for discussions. The ECC cylinders were attached with two dial gauges of 20 mm stroke length to determine the axial deformation. These deflection data were used further to compute the axial strain and to represent the compression stress versus strain behavior of fiber-reinforced engineered cementitious composite specimens.

### 3.3. Test Setup and Instrumentation Details

For achieving the dominant shear failure, all of the ECC specimens were investigated through a three-point bending test (Figure 1). During the testing, the specimens were loaded at a distance of 400 mm from one side of the support (i.e., shear-span) and not at the center of span length (L). This kind of test setup is provided to reduce the shear span (a_v_) to effective depth (d) ratio at one side and localize the shear failure. Hence, one side of the beam without stirrups had an a_v_/d ratio of 3.2 whereas the other part of the test side with stirrups had a high a_v_/d ratio of 4.8. The load was applied by means of a hydraulic jack with a maximum capacity of 500 kN in a load-controlled mode.

During testing, the load from the hydraulic jack was measured with the help of a load cell placed between the hydraulic jack and the spreader beam. The load from the hydraulic jack was transferred to the test specimens using the point loading arrangement (i.e., roller). At the point of load application, the beams were installed with the linear variable displacement transformer (LVDTs) and strain gauges to acquire the displacements and strains, respectively. The data found from the tests were recorded automatically with the help of the data acquisition system. In addition to the strain gauges, manual strain measurements were made on the other face of the test specimen using the demountable mechanical (DEMEC) strain gauges of 10 mm stroke and 100 mm gauge length. Small steel pellets were installed in a grid form close to the loading point to enable the manual strain measurement. The strain readings were taken when the loading was paused at every 1 kN interval.

## 4. Results and Discussions

### 4.1. Stress Versus Strain Behavior of ECC Specimen

**Figure 2:**
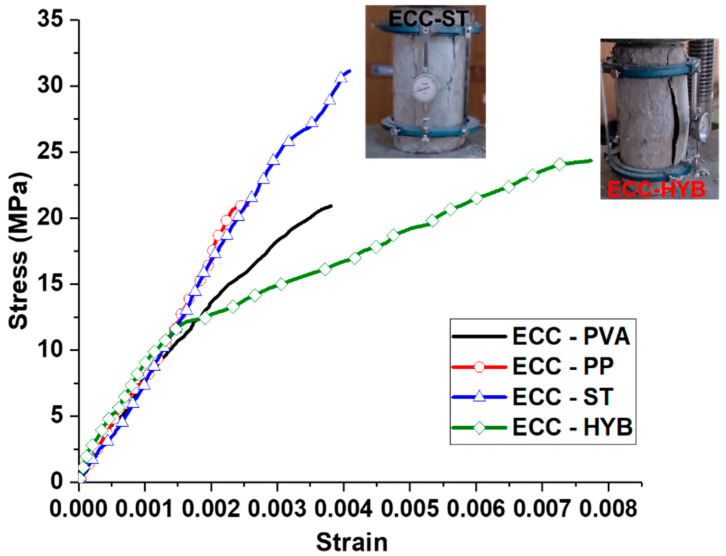
Compressive stress–strain behavior of ECC specimens.

Figure 2 demonstrates the compression stress versus strain behavior for ECC specimens with different fiber types. The outcomes of the compression test reveal that using a hybrid combination of PP and PVA fibers helped to significantly increase the failure strain of ECC specimens compared to the mono fiber ones [28]. ECC specimens with hybrid fibers had a mean cylinder compression strength of 24.3 N/mm^2^ and a large failure strain of 0.008. It is worth mentioning that the hybrid fiber addition resulted in a larger lateral expansion due to the effective lateral confinement effect [29,30]. Hence, the specimens had a large bulging in the lateral direction without sudden collapse. However, a similar failure type with large ductility was not found in the case of mono-fiber-reinforced ones. ECC specimens with steel fibers had an ultimate compressive strain and stress values of 0.0041 and 31.1 N/mm^2^, respectively. In spite of the larger compressive strength of steel-fiber-reinforced ECC specimens compared to hybrid ECC, the ultimate strain at failure was reduced by up to 100%, indicating the large ductility range induced by the hybrid combination of PVA and PP fibers [31]. Similarly, the ECC specimens with only PVA and PP fibers had a reduced ultimate strain of 0.0038 and 0.0031, respectively, compared to the hybrid ECC specimens.

**Figure 3:**
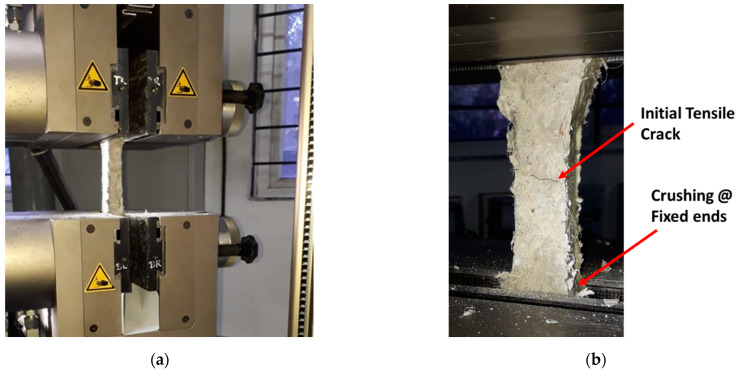
Tensile testing of ECC coupons. (**a**) Test Setup, (**b**) Failure Mode.

Similar to the compression tests, direct tension tests were conducted to demonstrate the strain-hardening characteristics of ECC. Dog-bone-shaped coupon samples were prepared with dimensions of 305 mm (length) × 65 mm (width) × 13 mm (thickness). The coupon samples were subjected to uniaxial tension loading in a servo-controlled universal testing machine (UTM), as depicted in Figure 3.

The results obtained from the tension test were not presented due to the inconsistency (i.e., grip failure and variation in peak loads). Nevertheless, the analytical framework developed by Suryanto et al. [32] based on the beam theory was used to determine ECC’s tensile stress–strain characteristics. For the casting of specimens, steel stirrups were used in the non-test zone. Coupon samples were prepared for both 10 mm longitudinal reinforcements and 8 mm stirrups and tested under axial tension using a 1000 kN computerized UTM. The reinforcements had an average yield stress, ultimate stress and rupture strain of 508 MPa, 610 MPa and 8.0%, respectively. The reported values of the steel tension test were found by testing at least three coupons.

### 4.2. Nonlinear Finite Element Analysis

A detailed nonlinear three-dimensional (3-D) finite element (FE) analysis was executed using the commercially available ABAQUS 6.13 software [33]. The FE model was created for reinforced ECC beams and the results were validated using benchmark experiments, including the load–deflection relationship, cracking and ultimate failure pattern. The validated FE model was further used for performing a detailed parametric investigation to develop design guidelines for the hybrid-fiber-reinforced ECC beams under shear.

#### 4.2.1. Material Modeling

**Figure 4:**
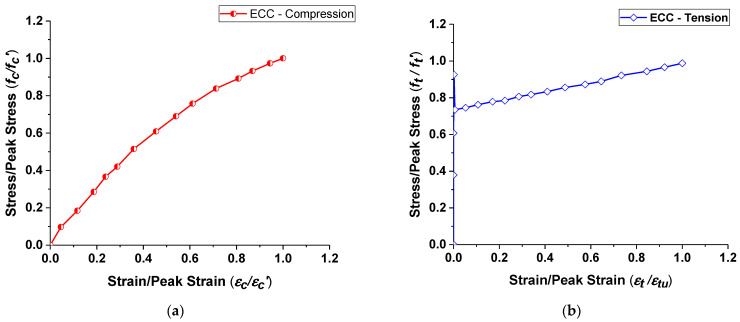

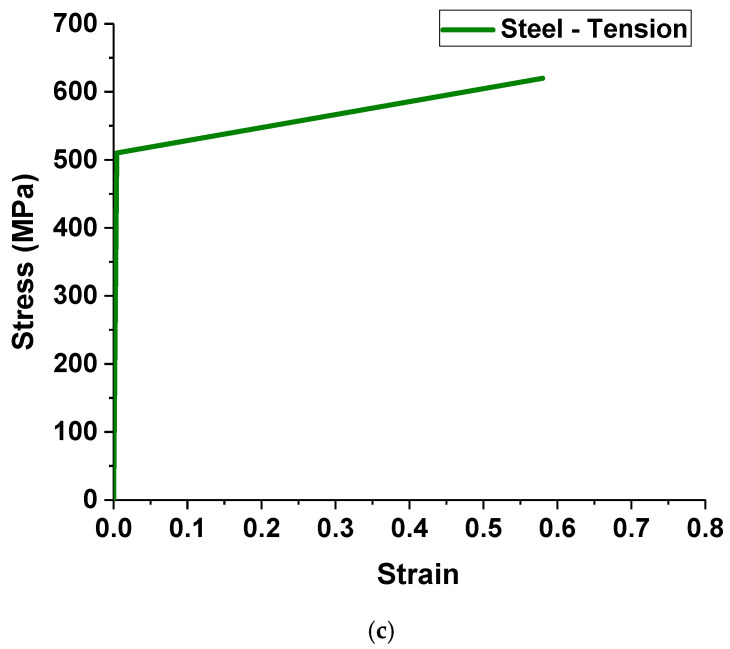
Material properties of ECC and steel rebars. (**a**) ECC in compression, (**b**) ECC in tension, (**c**) Steel reinforcement.

ECC is modeled using the concrete damage plasticity (CDP) model, which is an isotropic damage model and capable of predicting its nonlinear behavior under both compression and tension. The model recommended by Singh and Sivasubramanian [34] was used to determine ECC’s tension and compression curve, as shown in Figure 4. The tension stress–strain model was developed based on the experimental investigation of Kanda and Li [35]. Due to the high ductile nature of ECC, the model shows a large strain-hardening response after reaching the peak tensile stress. Assuming a particular strain value, the tensile stress can be estimated by Equations (1) and (2).

(1) $$\left(\frac{f_{t}}{f_{t}^{\prime }}\right)=134.75\left(\frac{\varepsilon_{t}}{\varepsilon_{tu}}\right)\;\mathrm{if}\;\left(\frac{\varepsilon_{t}}{\varepsilon_{tu}}\right)\;<\;0.00545$$

(2) $$\left(\frac{f_{t}}{f_{t}^{\prime }}\right)=0.2371\left(\frac{\varepsilon_{t}}{\varepsilon_{tu}}\right)+0.7334\;\mathrm{if}\;0.00545<\left(\frac{\varepsilon_{t}}{\varepsilon_{tu}}\right)<1$$

where $\varepsilon_{tu}$ = ultimate tensile strain; $f_{t}^{\prime }$ = ultimate tensile strength; $f_{t}$ = tensile stress in each loading; $\varepsilon_{t}$ = tensile strain in each loading.

Similarly, the compressive stress versus strain curve shown in Figure 4a is determined from the model proposed by Singh and Sivasubramanian [34]. The proposed model in compression was established from the experimental results of Li and Wang [36], where the ECC specimens exhibited a bi-linear behavior. Due to the high-ductile characteristics of ECC specimens, no softening post-peak behavior can be observed, which is common in the case of conventional concrete. The compression stress can be estimated using Equation (3) for a given strain value.

(3) $$\left(\frac{f_{c}}{f_{c}^{\prime }}\right)=1.64\left(\frac{\varepsilon_{c}}{\varepsilon_{cu}}\right)-0.65\left(\frac{\varepsilon_{c}}{\varepsilon_{cu}}\right)$$

where $\varepsilon_{cu}$ = compressive strain at ultimate condition; $\varepsilon_{c}$ = strain corresponding to particular load level; $f_{c}^{\prime }$ = peak compressive stress in (N/mm^2^); and $f_{c}$ = stress corresponding to particular load considered.

It is worth mentioning that the effect of different fibers can be incorporated into the FE model considering the ultimate compressive strength values from the cylinder test. The different plasticity parameters considered in the CDP model were inputted from the existing literature studies. The value of the dilation angle representing the angle obtained at the p–q plane at a large restraining pressure level was given as 53.2° for ECC. Moreover, the eccentricity (e) of the potential plastic surface was inputted as 0.10. The ratio of biaxial compressive yield stress (*f_b0_*) to initial uniaxial compressive yield stress (*f_c0_*) was given as 1.20. The CDP model uses the damage mechanisms to represent the damage intensity in the developed models. In the FE model, the damage parameters were inputted in a tabular form that varies from 0 to 1. These damage intensity factors correspond to the peak stress, where “1” corresponds to full damage and the value “0” signifies no damage. In the CDP model, the damage parameters corresponding to the inelastic strain were input. The inelastic strains in compression and tension can be obtained by subtracting the total strain from elastic strain as given in Equations (4) and (5), respectively.

(4) $$\varepsilon_{c}^{in}=\varepsilon_{c}^{}-\varepsilon_{0c}^{el}$$

where elastic strain in compression $\varepsilon_{0c}^{el}$ = σcE0, *E*_0_ = Young’s modulus and $\varepsilon_{c}^{}$ = total compressive strain.

(5) $$\varepsilon_{t}^{ck}=\varepsilon_{t}^{}-\varepsilon_{0t}^{el}$$

where elastic strain in tension $\varepsilon_{0t}^{el}$ = σtE0, *E*_0_ = Young’s modulus and $\varepsilon_{t}^{}$ = total tensile strain.

As shown in Figure 4c, the stress–strain behavior of steel was inputted through a bilinear stress–strain relationship. Here, the important parameters, such as yield stress, ultimate stress and failure strain, were inputted from the coupon test results.

#### 4.2.2. Modeling Procedure and Boundary Conditions

**Figure 5:**
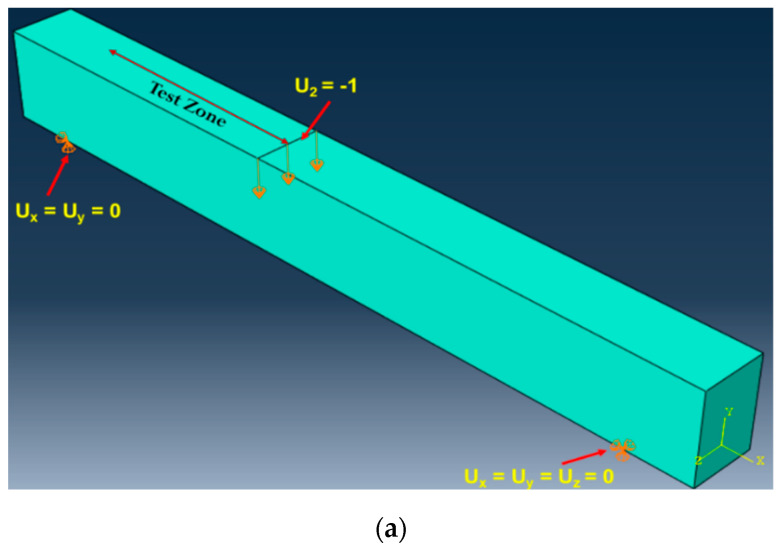

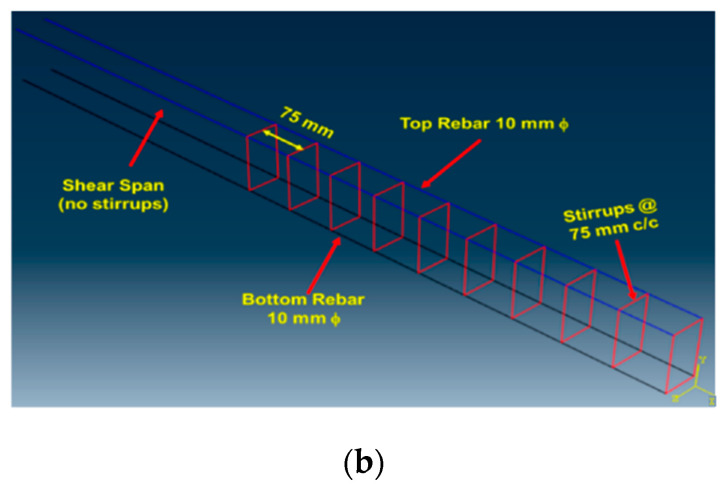
Representation of ECC and steel elements modelled in FE analysis: (**a**) concrete, (**b**) steel reinforcement.

For the FE modeling of ECC members, a dynamic explicit integration procedure was used. Though the load application was made under the static scenario, a dynamic explicit procedure was used to achieve a close convergence of overall predictions [37,38]. The interface amid the ECC and steel was modeled using the option “embedded-region”, which does not consider the bond-slip effect. For accurate boundary conditions, ECC beams were partitioned at the point of load application and supports. This partition helped to ensure the accuracy of load and support location, as depicted in Figure 5. All of the ECC members were modeled with the simply supported boundary condition, i.e., pinned support on one side and roller support on the other side. The loading was applied using the three-point bending configuration to simulate the actual test conditions. The load was applied through the incremental displacements, i.e., under the displacement-controlled mode, to effectively capture the post-peak behavior, which was not possible in the actual tests.

#### 4.2.3. Meshing and Mesh Convergence

Selecting a suitable mesh type could play a key role in achieving a reliable estimate of the overall predictions. ECC elements were developed using the C3D8R, the three-dimensional eight-node brick element with reduced integration. Each node of the C3D8R element has three translational degrees of freedom. A reduced integration scheme was used to improve the accuracy of predictions by eliminating the excessive stiffness due to shear locking. Steel reinforcements were modeled using the T3D2 element, a three-dimensional truss element containing two nodes. The mesh size was selected based on the results obtained from the mesh convergence study. Using a coarse mesh size of more than 30 mm yielded a higher value of FE predictions compared to the actual results. The size of the mesh was altered repeatedly to achieve close convergence. An optimum 20 mm mesh was used for the developed ECC specimens in the test matrix and parametric studies.

### 4.3. Load–Deflection Behavior Comparison

Table 3 displays the results obtained from the experiments and the comparison of the peak load with the predictions obtained from the FE analysis. No control beams were fabricated without discrete fibers to comply with the definition of ECC. Hence, the comparison of results was made based on the efficiency of mono and hybrid-fiber-reinforced ECC beams prepared using steel, PVA and PP fibers when compared with the performance of ECC beams with 2.0% micro polypropylene steel fibers. ECC beams with 2% micro PP fibers had an initial flexure crack at a load of 15.0 kN. The specimens attained a peak at a load and deflection of 34.1 kN and 15.8 mm, respectively. The beams failed due to the formation of diagonal shear–tension cracks. Similarly, for the ECC beams with 2% steel fibers, the initial flexure crack occurred at a 10.0 kN load, which is lower when compared to all of the other ECC specimens. On further loading, steel ECC beams attained a peak at a load and deflection of 37.6 kN and 9.4 mm, respectively. The beams failed due to a diagonal shear–tension mode with a higher load-carrying capacity of 10.3% compared to PP-based ECC beams. Comparing the performance of PVA-based ECC beams, the peak load-carrying capacity was approximately 55.0 kN, which is significantly higher (61.2%) than the polypropylene-fiber-reinforced ECC beams. The PVA ECC beam had an ultimate deflection of 13.1 mm and failed due to the shear–tension mode. Using hybrid PP and PVA fiber combinations yielded a better performance by enhancing ECC beams’ ultimate load and deflection. ECC beams with a 2% hybrid fiber combination had a peak load and displacement of 43.5 kN and 14.2 mm, respectively. The increase in peak load was 27.6% when compared to the ECC beams with 2% polypropylene fibers. Furthermore, the hybrid fiber combination changed the failure mode from brittle shear–tension to ductile flexure–shear, stopping the further spread of the shear fracture. Hence, comparing the four different series of specimens, hybrid ECC beams exhibited a better ductility response with a considerable enhancement in shear load capacity compared to the mono-fiber-reinforced ECC beams.

**Figure 6:**
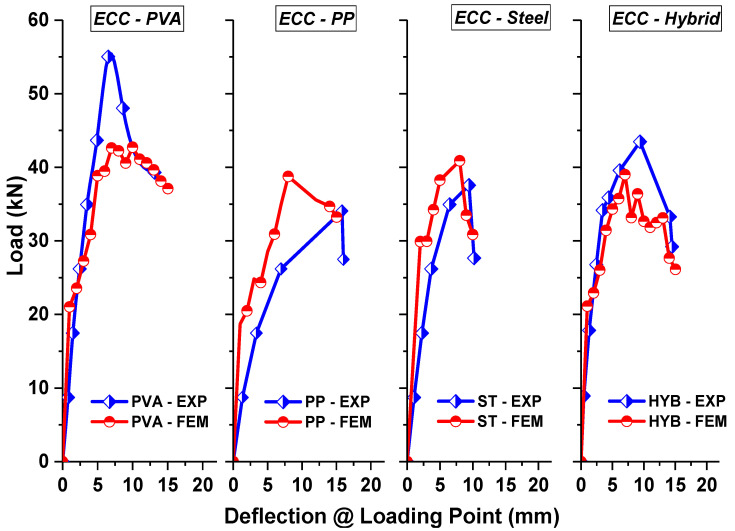
Load–deflection comparison for ECC specimens.

Figure 6 compares the load versus displacement obtained from the experimental and finite element predictions for different ECC beams. From the results, it can be understood that the overall behavior obtained from the FE analysis showed a good correspondence with the experiments in terms of the initial stiffness, post-cracking stiffness, peak strength and ultimate deflection limits. Moreover, the advantage of the developed FE model is the accuracy in predicting the post-peak response, which was not complete from the actual tests due to the limitations in the load-controlled testing mode. The average mean prediction ratio of peak strength between the finite element analysis (P_FEA_) and test results (P_EXP_) was found to be 0.98. This indicates that the average discrepancy of the ultimate load between the experimental and FE analysis was less than 10%, showing the reliability of the developed FE model.

### 4.4. Load–Strain Behavior and Energy Absorption Capacity

**Figure 7:**
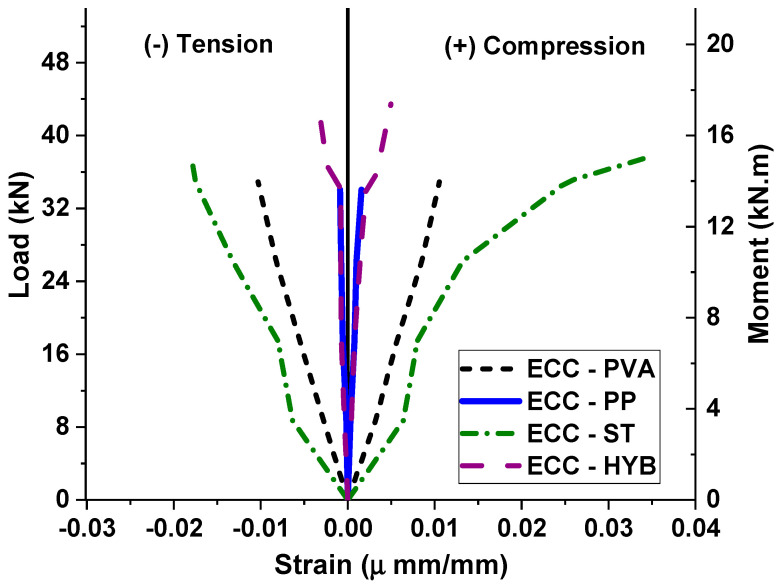
Load–strain behavior of ECC specimens with different fibers.

Figure 7 shows the load–strain behavior comparison for different ECC specimens under shear. The effectiveness of different fiber additions can be compared through their ability to slow down the intensity of damage progression, i.e., the strain levels in each specimen considering the same load value. Though the use of steel fibers showed a large value of tensile and compressive strain levels, the load corresponding to each strain was very low compared to other ECC specimens. The use of hybrid fibers showed a better performance by reducing the strain values at each load level until the value of the peak load. This reduction in strain values also indicates a reduced level of damage in the longitudinal reinforcements, thereby sustaining a large deformation capacity before ultimate failure.

**Figure 8:**
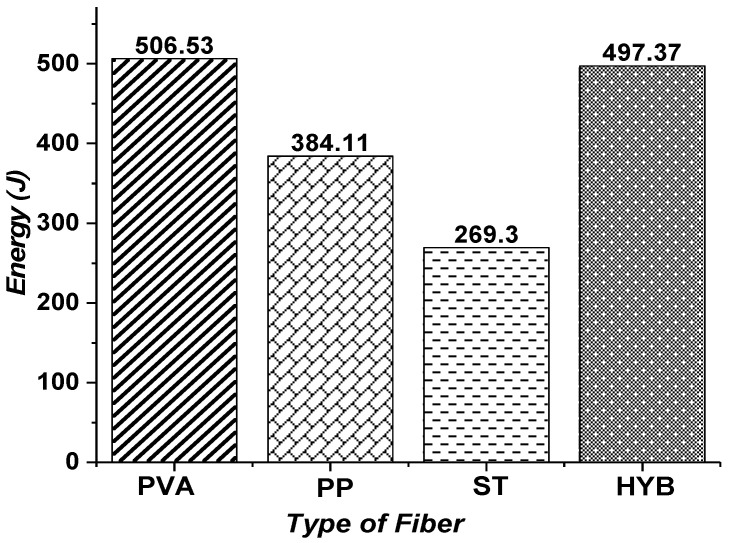
Energy absorption capacity of ECC beams with mono and hybrid fibers.

Figure 8 compares energy absorption capacity for mono and hybrid-fiber-reinforced ECC specimens under shear. Strain energy, also called the toughness or energy absorption capacity, is often used for accessing the effectiveness of fibers in shear resistance. The entire area behind the load-deflection diagram until the level of maximum deflection can be used to define toughness. Moreover, the point where the load has decreased by greater than 15% from the test specimen’s peak load is known as the ultimate deflection. The fiber addition could result in huge levels of energy dissipation due to various mechanisms such as de-bonding, pull-out and stretching. Hence, a large amount of energy has to be spent before the ultimate collapse. From the results, it is clear that the addition of hybrid fibers showed a large value of toughness when compared to the other mono-fiber-reinforced ECC members. The energy absorption capacity of hybrid ECC beams is 84.7% high when compared to the steel-fiber-reinforced ECC beams. Similarly, the micro PP and PVA-fiber-based ECC beams had 88.1% and 42.6% high energy absorption capacities when related to the steel-fiber-based ECC beams.

### 4.5. Crack Pattern and Failure Modes

**Figure 9:**
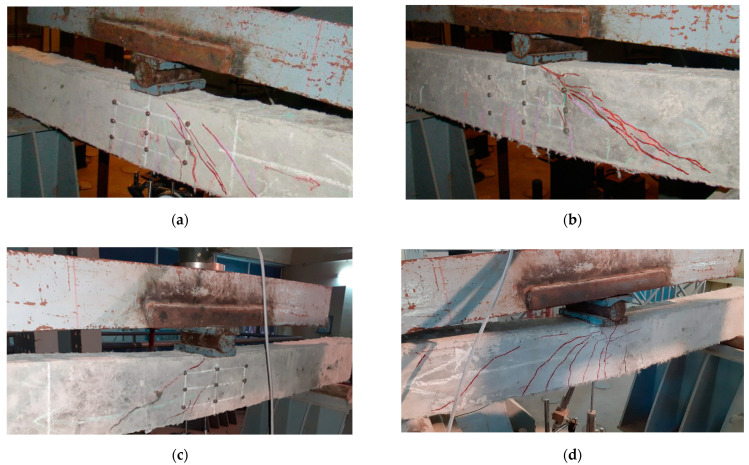

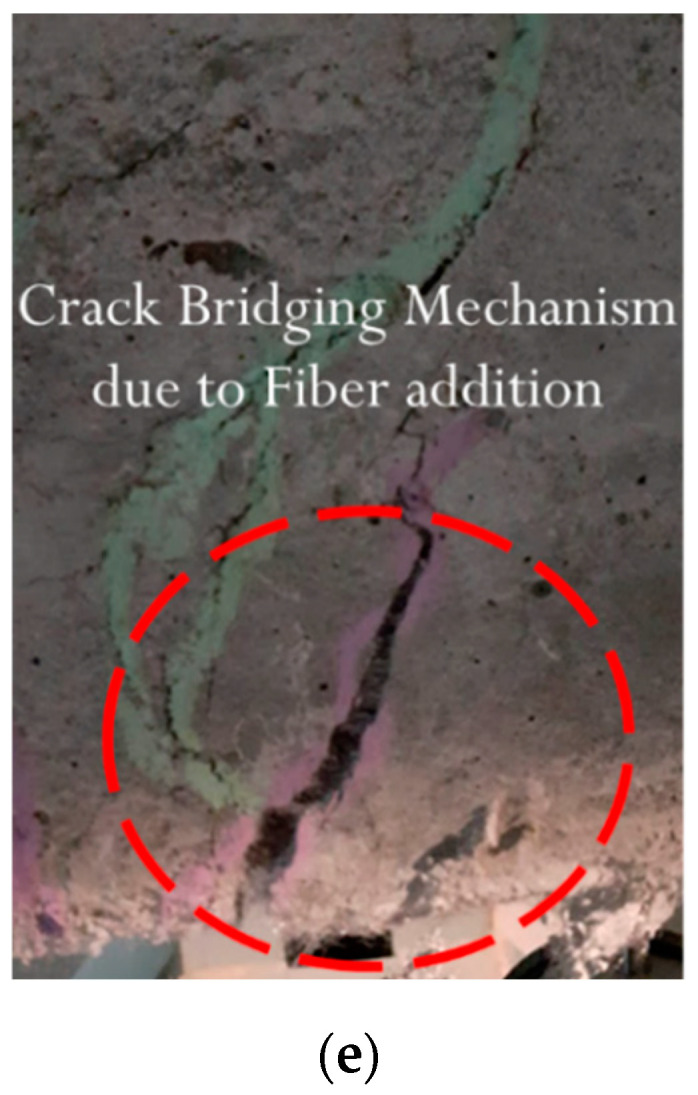
Experimental failure modes of ECC specimens with different fiber additions: (**a**) ECC—PVA, (**b**) ECC—PP, (**c**) ECC—ST, (**d**) ECC—HYB, (**e**) hybrid PVA + PP effects.

Figure 9 compares failure patterns for different ECC specimens under shear. All of the specimens had initial cracks due to flexure. On further load application, the shear cracks were developed in the shear span (i.e., test zone) and propagated to the support and loading point. ECC specimens with PVA fibers (ECC-PVA) failed due to the major shear crack opening. After the initial crack in flexure, a few more flexure cracks were observed on further loading. At the peak load, few flexure cracks were converted into flexure–shear cracks. Moreover, a primary shear crack occurred in the middle of the shear span and propagated towards the support and load point. This primary shear crack branched with the flexure–shear and other shear cracks at the ultimate load. Though the failure occurred because of the shear–tension type, the length of the major crack was measured to be less when compared to the other mono-fiber-reinforced ECC beams, i.e., the addition of PVA fibers increased the crack angle (θ = 58.9°). A similar failure mode was witnessed in the case of PP and steel-fiber-reinforced ECC beams. However, the length of the critical shear crack was high, i.e., the shear crack angle was found to be 35.9⁰ and 46.4⁰ for PP and steel-fiber-reinforced ECC beams, respectively, which portrays the ineffectiveness of PP and steel fibers in restraining the propagation of the major diagonal crack. The final failure occurred due to the combined flexure–shear mode for the ECC members with hybrid PVA and PP fibers. Hybrid ECC beams underwent failure initiation due to flexure cracks.

As depicted in Figure 9, multiple flexure cracking can be found in the shear span with the increase in load levels. At a load level of 75% of the peak load, a major diagonal crack (i.e., shear crack) was witnessed in the shear span. However, the hybrid fiber combination did not allow the shear crack to open and widen further. Hence, the flexure–shear crack in the shear span started to widen, as shown in Figure 9e. This flexure–shear crack propagated to the compression zone, where significant concrete crushing was witnessed. Hence, the failure of ECC beams was found to be more ductile by dissipating a large energy due to the fiber crack bridging mechanism compared to the other ECC beams with mono fibers. The inclination of a flexure–shear crack of ECC-HYB specimens was measured to be 68.5⁰ with respect to the longitudinal axis of the beam.

**Figure 10:**
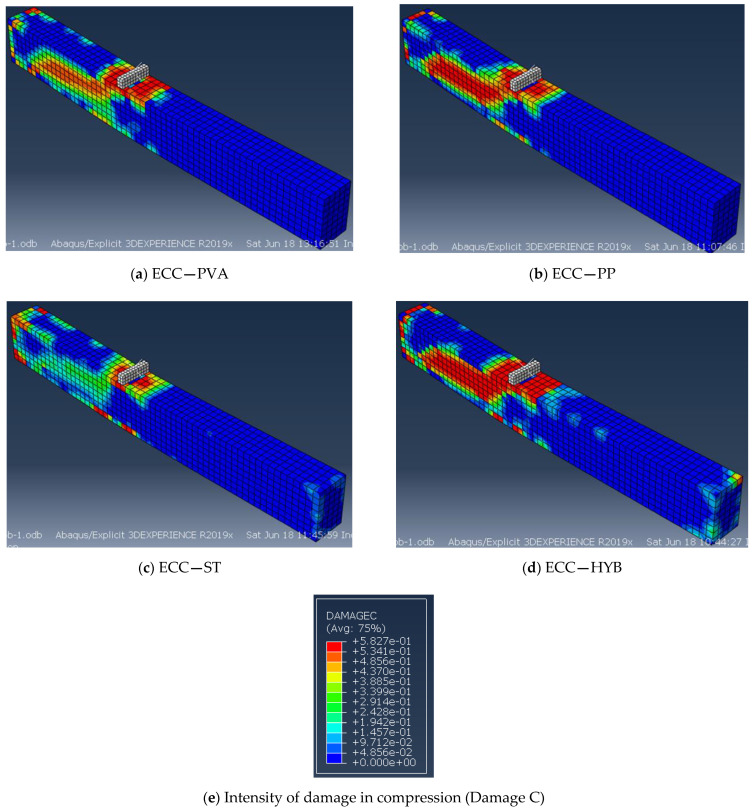
Crack pattern and failure mode of ECC specimens from FE analysis: (**a**) ECC—PVA, (**b**) ECC—PP, (**c**) ECC—ST, (**d**) ECC—HYB, (**e**) hybrid PVA + PP effects.

Figure 10 shows the failure pattern obtained from the FE analysis for the ECC beams with different fibers. The damage observed in the specimens is represented as damage parameters in compression and tension, as shown in Figure 10c. The represented damage index signifies the intensity of damage in ECC beams, which could vary from a maximum value of “1” to a minimum value of “0”. From the damage index, the value of “0” represents “no damage” and “1” represents the “failure or maximum damage”. The failure mechanisms discovered using FE analysis were consistent with the tests for all of the specimen series. All of the specimens had a shear–tension failure with the formation of S-type failure cracks at the ultimate deflection, as shown in Figure 10.

### 4.6. Parametric Studies

**Figure 11:**
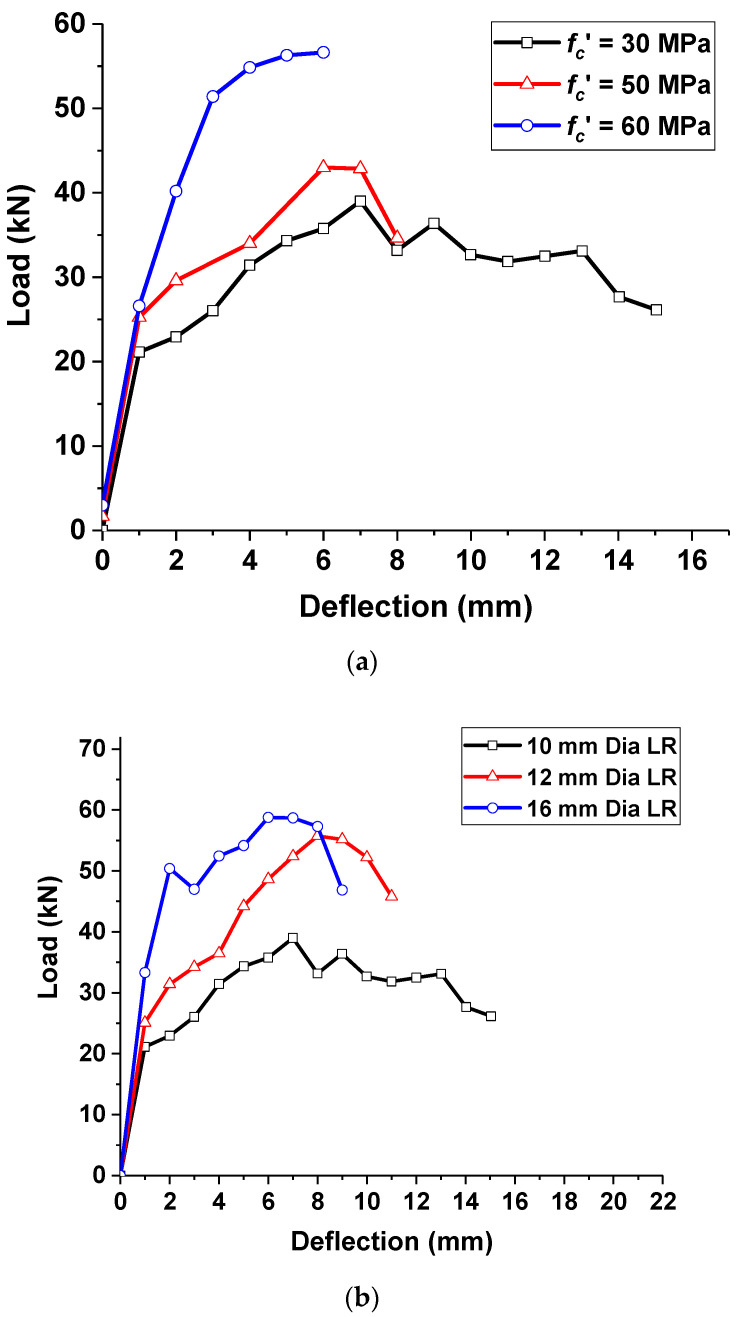
Parametric studies on the effect of strength and longitudinal reinforcement ratio: (**a**) effect of concrete strength and (**b**) effect of longitudinal reinforcement ratio.

The validated nonlinear FE model was used for performing a detailed parametric investigation. As this work emphasizes the shear performance of ECC specimens, the different parameters influencing their overall behavior were investigated. The different parameters investigated include (i) concrete strength, (ii) ratio of longitudinal bars and (iii) spacing of transverse reinforcements. In the first parametric study, three different concrete strengths were considered for hybrid ECC specimens: 30 MPa, 50 MPa and 60 MPa. From Figure 11a, it can be recognized that the compressive strength increase in ECC showed a good enhancement in the peak load with the reduction in ultimate deflection. For the ECC designed with a 30 MPa compressive strength, the maximum load-carrying capacity was 39.0 kN, corresponding to a deflection level of 6.99 mm. The compressive strength of ECC was 50 MPa and 60 MPa, and the shear load capacity increased by 10.3 and 43.6%, respectively, compared to the 30 MPa compressive strength of the ECC beam. However, the ultimate deflection limit was reduced considerably from 15.0 mm to 6.0 mm when the compressive strength of ECC increased to 60 MPa.

The effect of various longitudinal rebar ratios was investigated in the second parametric study. Three different sizes of longitudinal reinforcements—10 mm, 12 mm and 16 mm—were considered. From Figure 11b, it is found that the peak load capacity of ECC beams increased significantly with the increase in the reinforcement ratio. For the ECC beams with a 12 mm longitudinal reinforcement, the peak load capacity was found to be 55.7 kN, which is 42.8% higher than the ECC beam with a 10 mm diameter reinforcement.

Similarly, for the ECC beams with a 16 mm diameter longitudinal reinforcement, the peak load increased to 58.6 kN, which is 50.3% larger than the beams with a 10 mm diameter reinforcement. It is important to note that the increase in the load-carrying capacity of the beams due to the increment in longitudinal reinforcement ratio was due to the dowel action. The concrete contribution to shear showed a good enhancement. However, no significant increase in the load-carrying capacity was found when the reinforcement ratio was increased further due to the complete utilization of the shear contribution from dowel action.

The effect of transverse reinforcement spacing was investigated in the third parametric study. Originally, the beams tested in this work did not have any stirrups in the shear span due to the action of discrete fibers as a secondary reinforcement in arresting the shear cracks. Understanding the combined effect of fibers and transverse reinforcements in arresting the propagation of shear cracking is essential. Hence, two transverse reinforcement spacings were used to design stirrups in the shear span: 75 mm and 150 mm. The results of specimens with different stirrup spacings were compared with the control specimen with no stirrups.

**Figure 12:**
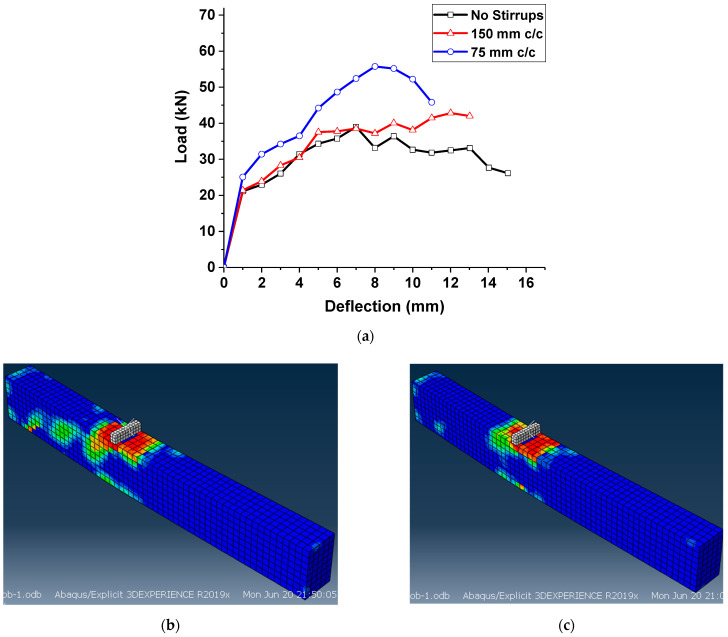
Parametric studies on hybrid ECC beams: (**a**) effect of transverse reinforcement spacing on shear span, (**b**) shear failure at 150 mm c/c stirrup spacing on shear span and (**c**) flexure failure at 75 mm c/c stirrup spacing on shear span.

From Figure 12a, it can be witnessed that the use of stirrups at a spacing greater than 150 mm did not yield any enhancement in the overall performance of hybrid-fiber-reinforced ECC beams. For the ECC beams with 150 mm c/c stirrup spacing, the peak strength was 42.8 kN, which was 9.7% higher than the control specimen. Moreover, the failure of specimens was due to the combined shear and flexure mode (Figure 12b), i.e., flexure–shear failure, where the yielding of longitudinal reinforcements can be observed. However, stirrups at a closer spacing of 75 mm helped to increase the shear resistance by 43.6% compared to the control specimen. Hence, from the above parametric study, it is clear that the provision of stirrups at a nominal spacing of 150 mm did not yield a significant enhancement in shear resistance due to the presence of hybrid fibers. Another important point is the conversion of failure from shear–tension type to bending mode due to the provision of a closer stirrup spacing of approximately 75 mm c/c.

## 5. Conclusions

This study investigated the shear behavior of mono and hybrid-fiber-reinforced engineered cementitious composite (ECC) beams. ECC beams with different fiber combinations were tested under an un-symmetrical three-point bending configuration at a low shear span (a_v_) to an effective depth (d) ratio of 3.2 to localize the shear failure to one side of the beam. In addition to the experimental studies, a detailed nonlinear finite element analysis was performed using the commercial software ABAQUS. The following major conclusions can be drawn from the present work

- The shear capacity of ECC beams with hybrid fibers was significantly higher when compared to other mono fiber combinations. ECC beams with 2% PVA fibers had the second best performance in peak load capacity and ultimate deflections. ECC beams with steel and PP fibers showed the lowest peak shear capacity.
- Adding hybrid fibers in ECC beams helped to increase the critical shear crack angle, which denotes the change in the type of failure from a brittle diagonal tension mode to a ductile bending mode. It is worth noting that adding other types of fibers was insufficient to change the failure mode.
- ECC beams’ energy absorption/toughness increased because of the use of hybrid PP and PVA fibers. Among the specimen series considered, hybrid fibers produced a higher energy absorption in ECC beams compared to other mono-fiber-reinforced ones.
- The predictions obtained from the FE analysis matched well with the experiments in terms of load versus deflection behavior and failure pattern. From the parametric investigation, it can be concluded that the use of stirrups at a closer spacing of 75 mm helped to enhance the shear resistance of ECC beams due to the combined contribution from fibers and steel reinforcement, thereby converting the failure type to flexure.
- The preliminary findings from the work on the shear behavior of hybrid-fiber-reinforced ECC beams provide insights into their effectiveness in enhancing the load-carrying capacity and conversion of the failure mode to ductile flexure mode. Hence, it can be concluded that the use of hybrid-fiber-reinforced ECC for the practical construction of shear dominant members may eliminate the need for the provision of transverse stirrups. However, a detailed study is further required to propose generic design guidelines for the practical implementation of hybrid-fiber-reinforced ECC members, which will be the scope for further work.

## Figures and Tables

**Table 1:** Properties of fibers used.

Parameters	Different Fibers Used		
Fiber Type	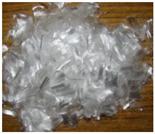	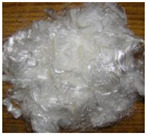	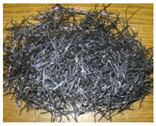
	Polypropylene	Polyvinyl Alcohol	Steel
Specific gravity	0.91	1.30	7.85
Tensile strength (MPa)	550	1600	1345
Elastic modulus (GPa)	3.5	39.0	200.0
Fiber length (mm)	20	12	30
Fiber diameter (mm)	0.022	0.040	0.5
Aspect ratio (L/D)	910	300	60

**Table 2:** ECC member details used.

Beam ID	Area of Tension Bars (mm^2^)	*ρ_s_* (%)	Type of Discrete Fiber	Fiber Content (*V_f_*)
ECC-ST	2Φ10 = 157	1.04	Steel	2.0%
ECC-PP	2Φ10 = 157	1.04	Polypropylene	2.0%
ECC-PVA	2Φ10 = 157	1.04	Polyvinyl Alcohol	2.0%
ECC-HYB	2Φ10 = 157	1.04	PVA + PP	2.0% (1.0% PVA + 1.0% PP)

Note: *ρ_s_* = longitudinal steel ratio on the tension side of the beam and *V_f_* = fiber volume fraction.

**Table 3:** Summary of test results.

Member ID	Initial Cracking Load (kN)	Initial Cracking Displ. (mm)	Ultimate Load (kN)	Displ. @ Ultimate Load	Energy Absorption (kN.mm)	Ultimate Load from FE (P_FEA_)	P_FEA_/P_EXP_ Ratio	Failure Mode	Angle of Critical Shear Crack (*θ_sc_*)
ECC-PVA	15.0	1.2	55.0	6.5	506.5	42.6	0.77	ST	58.8
ECC-PP	15.0	3.4	34.1	15.8	384.1	38.8	1.14	ST	35.9
ECC-ST	10.0	1.1	37.6	9.4	269.3	40.9	1.09	ST	46.4
ECC-HYB	15.0	1.3	43.5	14.2	497.4	39.0	0.90	FS	68.5

Note: FS—combined flexure–shear failure mode and ST—shear–tension failure.

## Data Availability

Not applicable.
